# Impact of Non-cardiac Comorbidities on Long-Term Clinical Outcomes and Health Status After Acute Heart Failure in China

**DOI:** 10.3389/fcvm.2022.883737

**Published:** 2022-07-13

**Authors:** Xiqian Huo, Lihua Zhang, Xueke Bai, Guangda He, Jiaying Li, Fengyu Miao, Jiapeng Lu, Jiamin Liu, Xin Zheng, Jing Li

**Affiliations:** National Clinical Research Center for Cardiovascular Diseases, State Key Laboratory of Cardiovascular Disease, Fuwai Hospital, National Center for Cardiovascular Diseases, Chinese Academy of Medical Sciences and Peking Union Medical College, Beijing, China

**Keywords:** heart failure, non-cardiac comorbidities, outcomes, quality of life, KCCQ

## Abstract

**Background:**

Individual non-cardiac comorbidities are prevalent in HF; however, few studies reported how the aggregate burden of non-cardiac comorbidities affects long-term outcomes, and it is unknown whether this burden is associated with changes in health status.

**Aims:**

To assess the association of the overall burden of non-cardiac comorbidities with clinical outcomes and quality of life (QoL) in patients hospitalized for heart failure (HF).

**Methods:**

We prospectively enrolled patients hospitalized for HF from 52 hospitals in China. Eight key non-cardiac comorbidities [diabetes, chronic renal disease, chronic obstructive pulmonary disease (COPD), anemia, stroke, cancer, peripheral arterial disease (PAD), and liver cirrhosis] were included, and patients were categorized into four groups: none, one, two, and three or more comorbidities. We fitted Cox proportional hazards models to assess the burden of comorbidities on 1-year death and rehospitalization.

**Results:**

Of the 4,866 patients, 25.3% had no non-cardiac comorbidity, 32.2% had one, 22.9% had two, and 19.6% had three or more in China. Compared with those without non-cardiac comorbidities, patients with three or more comorbidities had higher risks of 1-year all-cause death [heart rate, HR 1.89; 95% confidence interval (CI) 1.48–2.39] and all-rehospitalization (HR 1.35; 95%CI 1.15–1.58) after adjustment. Although all patients with HF experienced a longitudinal improvement in QoL in the 180 days after discharge, those with three or more non-cardiac comorbidities had an unadjusted 11.4 (95%CI −13.4 to −9.4) lower Kansas City Cardiomyopathy Questionnaire (KCCQ) scores than patients without comorbidities. This difference decreased to −6.4 (95%CI −8.6 to −4.2) after adjustment for covariates.

**Conclusion:**

Among patients hospitalized with HF in this study, a higher burden of non-cardiac comorbidities was significantly associated with worse health-related QoL (HRQoL), increased risks of death, and rehospitalization post-discharge. The findings highlight the need to address the management of comorbidities effectively in standardized HF care.

## Introduction

Heart failure (HF) is a major global public health concern, afflicting an estimated 64.3 million individuals worldwide and resulting in high morbidity and mortality (1). Notably, nearly 30% of the global increase in the number of HF cases has occurred in China during 1990–2017 (2). Non-cardiac comorbidities are prevalent in patients with HF and are usually associated with increased complexity of assessment and management (3). It was reported that nearly three-quarters of outpatients with chronic HF had one or more non-cardiac comorbidities in the European Heart Failure Pilot Survey (4), and more than 80% of Asian patients with HF had ≥2 comorbidities according to the ASIAN-HF registry (5). With the aging of the population worldwide, patients with comorbidities are becoming the norm rather than the exception.

Prior epidemiologic studies have reported the effect of individual non-cardiac comorbidities on preventable hospitalizations in chronic HF with reduced ejection (HFrEF) (6, 7) or on short-term outcomes (e.g., in-hospital death, 30-day death, or rehospitalization) in HF populations (8–13). Despite this, the effects of the overall burden of multiple non-cardiac comorbidities on long-term outcomes across HF ejection fraction groups are poorly understood in China. Furthermore, there is no prior evidence evaluating the association of the overall burden of non-cardiac comorbidities with patient-reported outcomes, including health-related quality of life (HRQoL) in acute HF. Understanding how non-cardiac comorbidities are distributed in individuals with HF in China and their overall prognostic associations with outcomes would be imperative to personalize an optimal treatment approach to improve health.

Accordingly, using data from a nationwide prospective cohort study among patients hospitalized for HF, we sought to: (1) characterize the patterns of non-cardiac comorbidities across ejection fraction groups in China; (2) assess the association between non-cardiac comorbidity overall burden and clinical outcomes; and (3) investigate the effects of non-cardiac comorbidity burden on quality of life (QoL) during their longitudinal experience.

## Methods

### Study Design and Participants

The rationale and design of the China Patient-centered Evaluative Assessment of Cardiac Events (PEACE)—Prospective Heart Failure Study have been described previously (14). In brief, we established a nationwide, multicenter prospective cohort of acute HF from 52 hospitals across 20 provinces, covering all economic-geographic regions in China. Patients were eligible if they were 18 years of age or older, local residents, and hospitalized with a primary diagnosis of new-onset HF or decompensated chronic HF determined by physicians. We consecutively screened eligible patients and sought written informed consent within 48 h of admission if they were willing to participate between August 2016 and May 2018. Eligible participants were interviewed during the index hospitalization and followed up at 1, 6, and 12 months after discharge. If a patient was unwilling or unable to attend the scheduled in-person interview, study information was obtained by telephone interviews with trained staff at the national coordinating center. We included patients discharged alive (*n* = 4,875) and further excluded those lost to follow-up at 1 year after discharge (*n* = 9, 0.02%).

The central ethics committee of Fuwai Hospital and local ethics committees of the participating hospitals approved the study. The study funder had no role in the study design, data collection, analyses and interpretation, or preparation of the manuscript. The study was registered at www.clinicaltrials.gov (NCT02878811).

### Non-cardiac Comorbidities

Eight key non-cardiac comorbidities were included in the analysis: diabetes mellitus (DM), chronic kidney disease (CKD), chronic obstructive pulmonary disease (COPD), anemia, stroke, cancer, peripheral arterial disease (PAD), and liver cirrhosis. DM was defined according to medical records or baseline laboratory results (i.e., HbA_1*C*_≥6.5%). CKD was defined according to discharge diagnosis on medical records or baseline laboratory results (estimated glomerular filtration rate (eGFR) <60 ml/min/1.73 m^2^) (15, 16). Anemia was defined according to medical records or baseline laboratory results (hemoglobin <120 g/L for men or <110 g/L for women). All other non-cardiac comorbidities (i.e., COPD, stroke, cancer, PAD, and liver cirrhosis) were classified based on medical record documentation (e.g., discharge diagnosis, the front page of medical record).

To describe the patterns of non-cardiac comorbidity burden among patients hospitalized for HF, patients were categorized into four groups according to the sum of non-cardiac comorbidities: none (no comorbidity), one, two, three, or more non-cardiac comorbidities as few patients had four or more non-cardiac comorbidities.

### Patient Characteristics

Data were collected *via* centralized medical chart abstraction, and interviews and physical examinations were conducted by trained local physicians following a standardized protocol. We set two levels of quality control measures to ensure the accuracy of data abstraction (14). Interviews were completed using a customized data collection system that employed real-time data entry validation to ensure the accuracy and completeness of data.

We collected patient demographics, socioeconomic status, clinical characteristics, laboratory biomarkers, medical treatments, and health status through central medical chart abstraction, patient interviews, and laboratory analysis. Age and sex were obtained as demographic information. Clinical characteristics included hypertension, elevated low-density lipoprotein cholesterol (LDL-C), smoking status, New York Heart Association (NYHA) class, left ventricular ejection fraction (LVEF), heart rate (HR), and systolic blood pressure (SBP) at admission, as well as evidence of prior cardiac diseases including HF, coronary heart disease (CHD), valve heart disease (VHD), and atrial fibrillation (AF). LDL elevation was defined as LDL-C ≥ 3.37 mmol/L (130 mg/dl) at admission. SBP and HR were measured using unified calibrated electronic devices. LVEF was measured according to standard echocardiogram protocol by trained physicians, and patients were categorized into HFrEF (LVEF ≤ 40%), HF with mildly reduced ejection fraction (HFmrEF, LVEF 41–49%), and HF with preserved ejection fraction (HFpEF, LVEF ≥ 50%) according to the updated 2021 ESC guideline (17). Laboratory tests at admission, including high-sensitivity cardiac troponin T (Hs-cTnT), N-terminal brain natriuretic peptide precursor (NT-pro BNP), serum creatine, blood urea nitrogen (BUN), and eGFR, were analyzed at a central laboratory using blood samples collected within 48 h, or local laboratory results if central tests were not available. Data on treatments at discharge were collected from medical records.

### Outcomes

Clinical outcomes of interest in this analysis included all-cause mortality and all-cause rehospitalization within 1 year of discharge. Rehospitalization was defined as the first unplanned admission to an inpatient unit or stay in an emergency department following discharge after the index hospitalization. Cardiovascular (CV) death was defined as sudden cardiac death, progressive HF death, death due to other CV causes (e.g., myocardial infarction, stroke, CV hemorrhage). HF rehospitalization was classified if the primary and contributing cause of rehospitalization was HF. The HF-specific health status outcome was 180-day QoL using the short version of the Kansas City Cardiomyopathy Questionnaire (KCCQ) (18), with scores ranging from 0 (worst) to 100 (best).

We ascertained outcome events using the same approach that we used in large international multicenter trials (19). We collected event information through follow-up interviews and the national database of death causes in China. An independent clinical event committee applied the protocol definitions and classified all-cause rehospitalizations and deaths occurring within 1 year of discharge. All data were sent to the national coordinating center for central adjudication by an independent clinical event committee.

### Statistical Analysis

We presented continuous variables as medians and interquartile range (IQR), and categorical variables as frequencies and percentages. To characterize the patterns of non-cardiac comorbidity burden across ejection fraction groups, we compared the prevalence of each non-cardiac comorbidity and the sum of comorbidities among patients with HFrEF, HFmrEF, and HFpEF using the Chi-squared test. We examined the differences in baseline characteristics across the sum of non-cardiac comorbidity categories using the Kruskal–Wallis test for trends in continuous variables (i.e., age, LVEF, HR, SBP, Hs-cTnT, NT-proBNP, creatinine, BUN, and eGFR), and Mantel–Haenszel tests for trends in categorical variables (all other variables).

We compared 1-year all-cause mortality and all-cause rehospitalization across the sum of non-cardiac comorbidity levels using the Kaplan–Meier analysis and log-rank tests. In multivariable models, we evaluated the effect of the sum of non-cardiac comorbidities on 1-year death and rehospitalization using frailty Cox proportional hazards models, accounting for patient clustering within hospitals. Candidate covariates in the multivariate model were selected based on a review of the literature and clinical experience, including age, sex, educational attainment, marriage, smoking, NYHA class, HR, SBP, LVEF, Hs-cTnT, NT-pro BNP, serum creatine, prior CHD, prior VHD, prior AF, and HF subtype (i.e., new-onset or decompensated chronic HF). We used a generalized linear regression model to evaluate the association of the sum of comorbidities and 180-day QoL using the KCCQ-12 score, both unadjusted and after adjusting for the above demographic and clinical risk profiles.

In this study, missing variables in the model (i.e., Hs-cTnT, NT-pro BNP, and LVEF) ranged from 0 to 6.0% and were suggestive of missing at random, and thus imputed using multiple imputation. A total of 1,165 (23.9%) patients were lacking the KCCQ data at 180 days due to death, loss to follow-up, or other reasons. To minimize the effects of selection bias, we performed the analysis using final complete cases analysis and then conducted a sensitivity analysis using probably inverse weighting on the probability of being observed.

We performed statistical analyses and graphs using SAS version 9.4 (SAS Institute, Cary, NC) and R software version 3.6.2 (R Foundation for Statistical Computing, Vienna, Austria). All comparisons were two-sided, and statistical significance was defined as *p* < 0.05.

## Results

### A Cohort Analysis and Non-cardiac Comorbidities Across Ejection Fraction Groups

A total of 4,866 patients were included in the final analysis. The median age of the cohort was 67 years (IQR, 57–75), and 1,826 (37.5%) were women. The mean number of non-cardiac comorbidities was 1.44 (±1.21). Overall, 25.3% of the whole population had no non-cardiac comorbidities, 32.2% had one, 22.9% had two, and 19.6% had three or more non-cardiac comorbidities. Among all non-cardiac comorbidities, DM was the most prevalent (31.7%), followed by CKD (31.3%), anemia (23.5%), and COPD (19.5%) (Table 1).

**TABLE 1:** Non-cardiac comorbidities by left ventricular ejection fraction (LVEF) groups.

	Total (*n* = 4866)	HFrEF (*n* = 1870)	HFmrEF (*n* = 1164)	HFpEF (*n* = 1832)	*P*-value
**Individual comorbidity**					
Diabetes mellitus	1541 (31.7)	560 (29.9)	395 (33.9)	586 (32.0)	0.067
COPD	948 (19.5)	304 (16.3)	199 (17.1)	445 (24.3)	<0.001
CKD	1525 (31.3)	527 (28.2)	395 (33.9)	603 (32.9)	<0.001
PAD	606 (12.5)	188 (10.1)	121 (10.4)	297 (16.2)	<0.001
Cancer	203 (4.2)	49 (2.6)	54 (4.6)	100 (5.5)	<0.001
Stroke	998 (20.0)	265 (14.2)	247 (21.2)	486 (26.5)	<0.001
Anemia	1145 (23.5)	313 (16.7)	297 (25.5)	535 (29.2)	<0.001
Liver cirrhosis	36 (0.7)	6 (0.3)	7 (0.6)	23 (1.3)	0.003
**Sum of non-cardiac comorbidities**					
None	1230 (25.3)	586 (31.3)	261 (22.4)	383 (20.9)	<0.001
One	1567 (32.2)	657 (35.1)	395 (33.9)	515 (28.1)	<0.001
Two	1116 (22.9)	390 (20.9)	276 (23.7)	450 (24.6)	<0.001
Three or more	953 (19.6)	237 (12.7)	232 (19.9)	484 (26.4)	<0.001

*COPD, chronic obstructive pulmonary disease; CKD, chronic kidney disease; PAD, peripheral arterial disease; HFrEF, HF with reduced ejection; HFmrEF, HF with mildly reduced ejection fraction; HFpEF, HF with preserved ejection fraction.*

In total, 1,870 (38.4%) patients were classified as HFrEF, 1,164 (23.9%) as HFmrEF, and 1,832 (37.6%) as HFpEF. Compared to patients with HFrEF and HFmrEF, the prevalence of non-cardiac comorbidity was significantly higher in patients with HFpEF with the exception of diabetes (*p* < 0.01). Patients with HFpEF had the highest number of non-cardiac comorbidities (1.68 ± 1.29), followed by HFmrEF (1.47 ± 1.17), while those with HFrEF had the lowest number of non-cardiac comorbidities (1.18 ± 1.09).

### Baseline Characteristics by the Burden of Non-cardiac Comorbidities

Table 2 presents baseline characteristics stratified by the burden of non-cardiac comorbidities. Compared with patients with a lower burden of non-cardiac comorbidities, those with a higher non-cardiac comorbidities burden were older and more frequently women (*p* < 0.001). The prevalence of hypertension, prior CHD, and prior HF was significantly higher in patients with a greater burden of non-cardiac comorbidities. Patients with a higher number of comorbidities were more likely to have higher levels of SBP, LVEF value, Hs-cTnT, NT-proBNP, BUN, and creatine at presentation; and were less likely to have β-blocker and aldosterone antagonists at discharge compared with the lower burden group (*p* < 0.001).

**TABLE 2:** Baseline characteristics by the burden of non-cardiac comorbidities.

	Total (*n* = 4866)	None (*n* = 1230)	One (*n* = 1567)	Two (*n* = 1116)	Three or more (*n* = 953)	*P*-trend
**Demographic**						
Age, yr (IQR)	67 (57, 75)	60 (50, 69)	65 (55, 74)	70 (62, 77)	74 (66, 79)	<0.001
Female (%)	1826 (37.5)	428 (34.8)	582 (37.1)	441 (39.5)	375 (39.3)	0.012
**Socioeconomic status (%)**						
Educational level						0.005
Primary school or below	2033 (41.8)	486 (39.5)	653 (41.7)	504 (45.2)	390 (40.9)	
Middle school	2175 (44.7)	566 (46.0)	723 (46.1)	466 (41.8)	420 (44.1)	
College or above	517 (10.6)	143 (11.6)	153 (9.8)	107 (9.6)	114 (12.0)	
Married	3817 (78.4)	1008 (82.0)	1235 (78.8)	839 (75.2)	735 (77.1)	<0.001
**Clinical risk factors**						
Current smoker (%)	1005 (20.7)	379 (30.8)	422 (26.9)	251 (22.5)	168 (17.6)	<0.001
Hypertension (%)	2843 (58.4)	511 (41.5)	858 (54.8)	735 (65.9)	739 (77.5)	<0.001
LDL-C elevation (%)	746 (15.3)	185 (15.0)	253 (16.1)	177 (15.9)	131 (13.7)	0.438
NYHA (%)						0.036
I-II	704 (14.5)	210 (17.1)	215 (13.7)	166 (14.9)	113 (11.9)	
III	2155 (44.3)	533 (43.3)	698 (44.5)	475 (42.6)	449 (47.1)	
IV	2007 (41.2)	487 (39.6)	654 (41.7)	475 (42.6)	391 (41.0)	
LVEF, % (IQR)	43 (33, 56)	40 (30, 53)	42 (31, 54)	44 (34, 58)	49 (38, 60)	<0.001
HR, beats/min (IQR)	86 (74, 100)	89 (75, 104)	87 (75, 101)	86 (73, 100)	84 (72, 96)	<0.001
SBP, mmHg (IQR)	130 (116, 148)	126 (111, 140)	130 (115, 147)	132 (118, 150)	137 (120, 153)	<0.001
**Cardiac medical history (%)**						
CHD	2815 (57.9)	537 (43.7)	846 (54.0)	704 (63.1)	728 (76.4)	<0.001
HF	3408 (70.0)	815 (66.3)	1075 (68.6)	804 (72.0)	714 (74.9)	<0.001
VHD	793 (16.3)	229 (18.6)	257 (16.4)	162 (14.5)	793 (16.3)	0.012
AF	1771 (36.4)	440 (35.8)	569 (36.3)	416 (37.3)	346 (36.3)	0.658
**Electrocardiogram (ECG) (%)**						
QRS ≥ 120 ms	867 (17.8)	230 (18.7)	285 (18.2)	187 (16.8)	165 (17.3)	0.255
**Laboratory test**						
Hs-cTnT, ng/L (IQR)	22 (13, 44)	17 (10, 32)	21 (12, 39)	24 (15, 46)	33 (18, 69)	0.010
NT-proBNP, pg/ml (IQR)	1547 (628, 3536)	1213 (495, 2571)	1445 (638, 3118)	1676 (635, 4046)	2311 (831, 6000)	<0.001
eGFR, ml/min/1.73 m^2^ (IQR)	73 (57, 88)	83 (73, 95)	76 (64, 91)	66 (53, 84)	52 (37, 65)	<0.001
BUN, mmol/L (IQR)	7 (5, 9)	6 (5, 7)	7 (5, 8)	7 (5, 10)	9 (6, 12)	<0.001
Creatinine, μmol/L (IQR)	93 (78, 112)	84 (74, 95)	89 (77, 104)	97 (79, 121)	120 (99, 156)	<0.001
**Medications at discharge (%)**						
Digoxin	1168 (24.0)	329 (26.7)	398 (25.4)	254 (22.8)	187 (19.6)	<0.001
Beta-blocker	2870 (59.0)	780 (63.4)	950 (60.6)	605 (54.2)	535 (56.1)	<0.001
ACEI/ARB	2537 (52.1)	665 (54.1)	827 (52.8)	582 (52.2)	463 (48.6)	0.014
Aldosterone antagonists	3091 (63.5)	818 (66.5)	1034 (66.0)	687 (61.6)	552 (57.9)	<0.001
Diuretics	3355 (68.9)	813 (66.1)	1101 (70.3)	759 (68.0)	682 (71.6)	0.520
**Quality of life, mean ± SD**						
KCCQ at baseline	44.1 ± 22.7	48.2 ± 22.0	45.1 ± 22.7	42.1 ± 22.4	39.4 ± 22.8	<0.001
KCCQ at 180 days	72.9 ± 20.8	77.1 ± 18.2	74.8 ± 19.8	70.8 ± 21.5	65.7 ± 23.0	<0.001

*IQR, interquartile range; LDL-C, low-density lipoprotein cholesterol; NYHA, New York Heart Association; LVEF, Left Ventricular Ejection Fraction; HR, heart rate; SBP, Systolic Blood Pressure; CHD, Coronary Heart Disease; HF, heart failure; VHD, valve heart disease (VHD); AF, atrial fibrillation; Hs-cTnT, high sensitivity cardiac troponin T; NT-pro BNP, N-terminal brain natriuretic peptide precursor; eGFR, estimated glomerular filtration rate; BUN, blood urea nitrogen; ACEI, angiotensin-converting enzyme inhibitor; ARB, angiotensin-receptor blocker; KCCQ, Kansas City Cardiomyopathy Questionnaire.*

### Association of Non-cardiac Comorbidities With All-Cause Mortality and All-Cause Rehospitalization After Heart Failure

**FIGURE 1:**
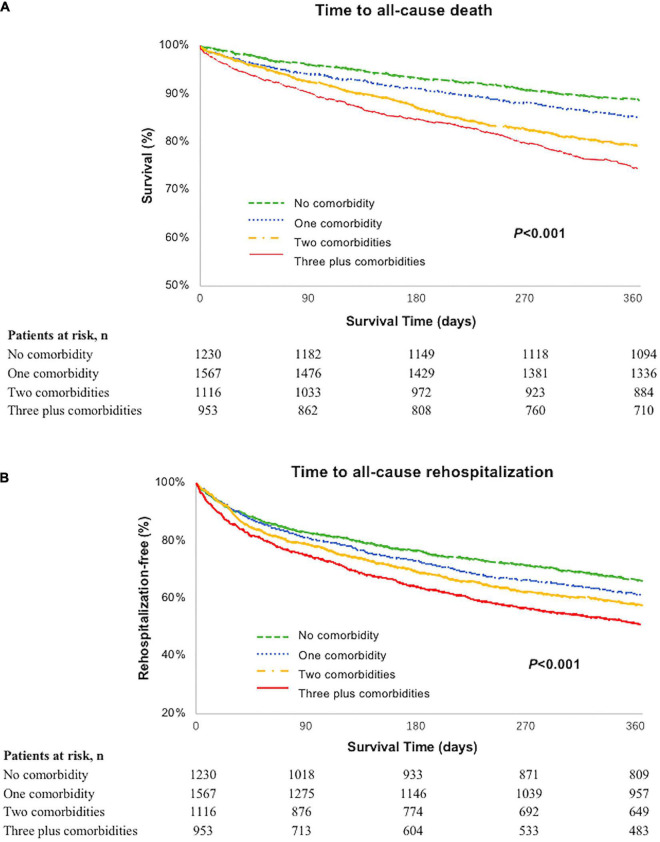
**(A)** Unadjusted Kaplan–Meier curve: 1-year all-cause mortality by burden of non-cardiac comorbidities; **(B)** 1-year all-cause rehospitalization by burden of non-cardiac comorbidities.

Groups with the highest comorbidity burden had significantly greater unadjusted rates of 1-year all-cause mortality and all-cause rehospitalization compared with the lower burden group, as shown in Figures 1A,B. In particular, of patients with three or more non-cardiac comorbidities, 245 (25.7%) died within 1 year compared with 138 (11.2%) in those without non-cardiac comorbidity (*p* < 0.001). Within 1 year of discharge, nearly half of the patients with ≥3 non-cardiac comorbidities had an all-cause rehospitalization (*n* = 470, 49.3%) compared with 34.5% (*n* = 424) of the patients without any non-cardiac comorbidity (*p* < 0.001), with the divergence of the curves occurring approximately 1 month after discharge.

**FIGURE 2:**
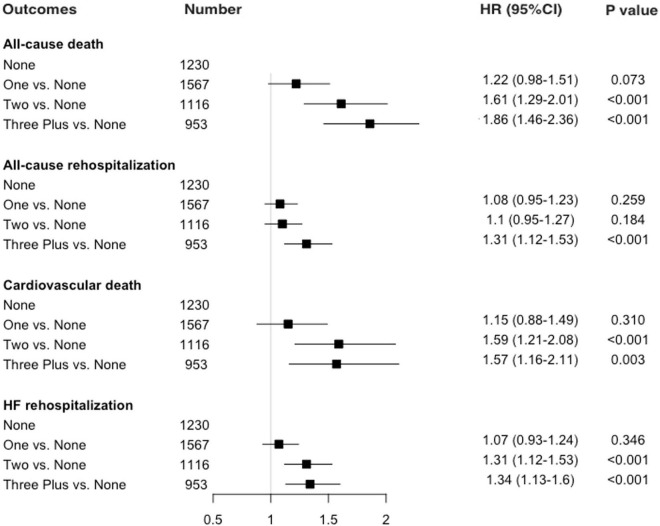
Adjusted association between non-cardiac comorbidities and 1-year mortality and rehospitalization.

In the multivariate model, three or more non-cardiac comorbidities were independently associated with an 86% higher risk of 1-year all-cause mortality [HR 1.86; 95% confidence interval (CI) 1.46–2.36] after adjusting for demographic and risk profiles (Figure 2). For all-cause rehospitalization, patients with ≥3 non-cardiac comorbidities were more likely to be readmitted within 1 year of discharge than those without comorbidities (HR 1.31; 95%CI 1.12–1.53). We observed no significant relationship between one or two non-cardiac comorbidity and all-cause rehospitalization after adjustment.

### Association of Non-cardiac Comorbidities With Cardiovascular Mortality and Heart Failure Rehospitalization

The effects of comorbidity burden on 1-year CV death and HF rehospitalization risk are shown in Figure 2. The patterns were similar for CV death, which accounted for about 86% of all deaths within 1 year of discharge. Patients with ≥3 non-cardiac comorbidities had a 57% increase of 1-year CV death risk as compared with those with no non-cardiac comorbidities (HR 1.57; 95%CI 1.16–2.11) after adjustment, though the non-significant association was observed between patients with one vs. no comorbidities. Similarly, in the multivariable model, the group with three or more comorbidities was associated with an increased risk of 1-year HF rehospitalization risk (HR 1.34; 95%CI 1.13–1.60) than the group without non-cardiac comorbidities. Individual non-cardiac comorbidity on outcomes is demonstrated in Supplementary Table 1.

### The Burden of Non-cardiac Comorbidities and Quality of Life in Heart Failure

We explored longitudinal changes in QoL among patients with HF and non-cardiac comorbidities. Overall, a comparison of patients who reported QoL (i.e., KCCQ) data at 180 days with those who did not reveal no significant differences in major demographic and clinical characteristics that might bias the results.

At baseline, compared with those without non-cardiac comorbidities, patients with a greater burden of non-cardiac comorbidities had lower KCCQ scores, with the largest disparities of −8.8 points between the groups with three or more comorbidities and without comorbidities (39.4 ± 22.8 vs. 48.2 ± 22.0, *p* < 0.001; Table 2); however, after the adjustment for covariates, the disparities attenuated to − 5.4 points but remained significant (95% CI − 7.4 to − 3.4, *p* < 0.001). Those with two or one non-cardiac comorbidities had 3.5 (95%CI −5.3 to −1.7, *p* < 0.001) and 1.6 (95%CI −3.1 to −0.0, *p* = 0.052) adjusted KCCQ scores lower than patients without non-cardiac comorbidities.

In the 180 days after discharge, all patients with and without non-cardiac comorbidities experienced a longitudinal improvement in their QoL (Table 1). The mean score in patients with three or more non-cardiac comorbidities was 65.7 ± 23.0, compared with 77.1 ± 18.2 among those with no comorbidities at 180 days. Patients with three or more non-cardiac comorbidities had unadjusted 11.4 (95%CI −13.4 to −9.4, *p* < 0.001) scores lower than those without comorbidities. After accounting for covariates, patients with ≥3 comorbidities had significantly 6.4 (95%CI −8.6 to −4.2, *p* < 0.001) adjusted KCCQ scores lower than those without comorbidities (Table 3).

**TABLE 3:** Effect of the burden of non-cardiac comorbidities on quality of life (QoL).

	HF-specific KCCQ summary score			
	Baseline		6 month	
	Unadjusted	Adjusted*	Unadjusted	Adjusted*
None	0 (ref)	0 (ref)	0 (ref)	0 (ref)
One vs. none	−3.1 (−4.8,-1.4)	−1.6 (−3.1, 0.0)	−2.3 (−4.2,-0.6)	−0.5 (−2.2, 1.1)
Two vs. none	−6.1 (−7.9, −4.3)	−3.5 (−5.3, −1.7)	−6.3 (−8.2, −4.4)	−2.9 (−4.9, −1.0)
Three plus vs. none	−8.8 (−10.7, −6.9)	−5.4 (−7.4, −3.4)	−11.4 (−13.4, −9.4)	−6.4 (−8.6, −4.2)

*Adjusted for age, sex, educational attainment, marriage, smoking, NYHA class, HR, SBP, LVEF, Hs-cTnT, NT-pro BNP, serum creatine, prior CHD, prior VHD, and prior AF. KCCQ, Kansas City Cardiomyopathy Questionnaire.

To address concerns about missing QoL data, we used a propensity model to generate a predicted probability to be the missing score. We used the reciprocal of this score to weigh patients with missing QoL data in multivariable regression models identical to the frailty models but without hierarchical modeling. The propensity score models produced comparable results (Supplementary Table 2).

## Discussion

In this first comprehensive report on the overall burden of non-cardiac comorbidities and their association with outcomes and health status in a national cohort of patients with acute HF in China, we found that non-cardiac comorbidities are highly prevalent, and patients with ≥3 non-cardiac comorbidities had a 90% higher risk of 1-year all-cause mortality and a 40% higher risk of all-rehospitalization compared with those without comorbidities after adjustment. Although QoL in patients with HF improved after discharge, patients with ≥3 non-cardiac comorbidities had significant 11.4 lower KCCQ scores than those without comorbidities.

In this study, about three-quarters of patients hospitalized for HF were reported to have one or more non-cardiac comorbidities in China, the prevalence of which was comparable with that of the ESC-HF Pilot Study (4) but lower than that of the US cohort of elderly HF patients in the GWTGHF registry (9). The higher burden of comorbidities in HFpEF was supported by previous HF registries and trials in western populations (20–22). However, we are not aware of any nationwide studies on Chinese adults that have provided data on patterns of the overall burden of comorbidities in HF across the whole spectrum of systolic function. Moreover, the substantial lack of data on numerical ejection fraction in prior studies allowed only for comparisons between those with LVEF ≥40% and <40%, potentially mischaracterizing or leaving out those with HFmrEF (8). With regard to generalizability, our study cohort included a population consecutively enrolled from 52 hospitals covering all geographic-economic regions in China, and represented substantial diversity in patient characteristics, medical resource allocation, and the quality of medical care. Although older and more severe patients had fewer opportunities to be enrolled in the cohort, we still enrolled a large number of these patients. One-fifth of the patients were older than 75 years, and two-fifths were in NYHA class IV. Considering the management complexity and lack of proven therapies in HFpEF, the prevention and treatment of comorbidities would remain a mainstay of therapy for this population.

This study also expands on the literature by providing an insight into the overall burden of non-cardiac comorbidities and long-term outcomes in acute HF. Most of the published studies showed that individual comorbidity in HF, such as diabetes, CKD, COPD, or anemia, can be independently associated with higher risks of adverse outcomes (10–13, 23); however, few studies explored the cumulative prognostic effects of non-cardiac comorbidity burden on outcomes. Assessing the possible association based on individual disorders may be subject to a collider bias, leading to distorted results. Moreover, prior studies mainly focused on short-term outcomes including 30-day mortality/HF rehospitalization and in-hospital mortality (8, 9), making it difficult to draw conclusions on long-term outcomes. Our analysis found a significant association between non-cardiac comorbidity burden and the risk of death and/or hospitalization at 1 year, especially the risk of HF rehospitalization. Potential explanations for these associations include that non-cardiac comorbidities can affect cardiac structure and function, contribute to changes in HF pathophysiology, alter the natural history of HF, and increase the risks of rehospitalizations and mortality (24). Additionally, understanding the pattern of comorbidities and their association with outcomes is particularly important in China, which is experiencing a growing burden of HF and comorbidities. Evidence from Western countries is unlikely to generalize to the unique disease patterns of China.

To the best of our knowledge, this is the first analysis to assess the association between the *overall* burden of non-cardiac comorbidities and HRQoL in patients with HF. In addition to survival, patients with HF are especially concerned about their QoL. The present analysis showed that patients with a higher non-cardiac comorbidities burden tended to have worse QoL. According to previous studies, a mean change in the range of 3.6–5.0 points in KCCQ-12 summary scores is considered clinically significant (25). Dr. Van den Berge’s study previously compared QoL among patients with or without any comorbidities in HF, but they did not assess the impact of the overall burden of non-cardiac comorbidities on health status, and the study conclusion was limited to a relatively small sample size of 334 patients who had completed HRQoL questionnaires at baseline (26). It is plausible that patients with a higher burden of coexisting comorbidities may experience worse QoL due to therapeutic complexity, and increased financial burden in multimorbidity management. In 2019, the US Food and Drug Administration reinforced that therapies safely improve patient-reported outcomes, such as HRQOL, may meet regulatory approval standards in HF (27). Despite the broad recognition of HRQOL as an important outcome, insights regarding optimized comorbidity management in routine HF care to improve QoL are lacking. Our findings are important for clinicians and healthcare policymakers dedicated to initiating comorbidities prevention and treatment programs after hospitalizations for HF.

Heart failure is a rapidly growing and costly clinical syndrome that imposes a massive burden on mortality and morbidity. Concomitant non-cardiac conditions, including renal disorders, chronic respiratory diseases, and diabetes, are highly prevalent and prognostically important in patients diagnosed with HF. Current guidelines in HF focus mainly on improving CV status; however, holistic, patient-centered care with optimal management of the most prevalent comorbidities also needs to be included in HF guideline-driven practice to improve survival. Our findings support a shift toward an expanded care model that considers the impact of concomitant diseases on impaired outcomes, and then determines appropriate treatment strategies in HF management. A better understanding of the factors that contribute to the effects of comorbidity burden on health outcomes after HF is also warranted.

The results of our analysis have several limitations. First, the data in this study are observational and there may exist potential selection bias and unobserved confounders (e.g., psychological factors, health literacy) that explain the relationship. We acknowledge that this is an intrinsic limitation to observational studies. Second, we realize that non-cardiac comorbidities included in our analyses are not exhaustive; however, the selection of non-cardiac comorbidities in our study was based on literature review and author consensus. Third, although this study was conducted in selected hospitals that represent many geographic regions, the results may not be generalizable to the whole population in China. However, the overall distribution of comorbidity burden, as well as the outcomes among those with HF, is consistent with those observed in national data from China and other countries. Finally, approximately one-fourth of the cohorts did not report QoL data at 180 days. However, we conducted sensitivity analyses with both complete cases and with a probably inverse weighted approach and found concordant results.

## Conclusion

Non-cardiac comorbidities are prevalent in patients hospitalized for HF in China. Greater burden of non-cardiac comorbidities was associated with increased risks of 1-year mortality and rehospitalization, as well as worse QoL in HF. Further efforts to optimize the prevention and management of non-cardiac comorbidities in HF would be crucial for this vulnerable patient group.

## Data Availability Statement

The original contributions presented in this study are included in the article/Supplementary Material, further inquiries can be directed to the corresponding author.

## Ethics Statement

The studies involving human participants were reviewed and approved by the Central Ethics Committee at Fuwai Hospital and local ethics committees at participating hospitals approved the study. The patients/participants provided their written informed consent to participate in this study.

## Author Contributions

JL conceived and designed the study. XH drafted the manuscript with LZ. XB analyzed the data as the statistician. FM, GH, JML, JPL, JYL, and XZ revised the manuscript for important intellectual content. All authors participated in the interpretation of the data and approved the final version of the manuscript.

## Conflict of Interest

JL was the recipient of research grants from the government of China, through Fuwai Hospital, for research to improve the management of hypertension and blood lipids, and to improve care quality and patient outcomes of cardiovascular disease, a recipient of research agreements with Amgen, through National Center for Cardiovascular Diseases (NCCD) and Fuwai Hospital, for a multi-center trial that assesses the efficacy and safety of Omecamtiv Mecarbil, and for dyslipidemic patient registration, recipient of a research agreement with Sanofi, through Fuwai Hospital, for a multi-center trial on the effects of sotagliflozin, recipient of a research agreement with University of Oxford, through Fuwai Hospital, for a multi-center trial of empagliflozin, and also was a recipient of a research agreement, through NCCD, from AstraZeneca for clinical research methods training from Lilly for physician training outside the submitted work. The remaining authors declare that the research was conducted in the absence of any commercial or financial relationships that could be construed as a potential conflict of interest.

## Publisher’s Note

All claims expressed in this article are solely those of the authors and do not necessarily represent those of their affiliated organizations, or those of the publisher, the editors and the reviewers. Any product that may be evaluated in this article, or claim that may be made by its manufacturer, is not guaranteed or endorsed by the publisher.
