# Supplementary material for: Impact of Non-cardiac Comorbidities on Long-Term Clinical Outcomes and Health Status After Acute Heart Failure in China
Source: Front Cardiovasc Med. 2022 Jul 13;9:883737. doi: 10.3389/fcvm.2022.883737 (PMC9326097; doi:10.3389/fcvm.2022.883737)
Supplement: Supplementary file 1 [file Data_Sheet_1.docx]

SUPPLEMENTAL MATERIAL

**TABLE OF CONTENTS**

**eTable1 Analysis of individual non-cardiac comorbidities and association with outcomes**

**eTable2 Sensitivity analysis using IPW to assess the effect of the burden of non-cardiac comorbidities on quality of life.**

**eTable 1. Analysis of individual non-cardiac comorbidities and association with outcomes**

| **Non-cardiac comorbidity** | **Unadjusted**  **HR (95% CI)** | ***P* value** | **Adjusted***  **HR (95% CI)** | ***P* value** |
| --- | --- | --- | --- | --- |
| **1 year all-cause mortality** |  |  |  |  |
| Diabetes mellitus | 1.16 (1.0-1.34) | 0.044 | 1.18 (1.02-1.37) | 0.029 |
| CKD | 1.80 (1.56-2.06) | <0.001 | 1.26 (1.08-1.45) | 0.002 |
| COPD | 1.39 (1.18-1.64) | <0.001 | 1.29 (1.09-1.53) | 0.003 |
| Anemia | 1.95 (1.69-2.25) | <0.001 | 1.55 (1.34-1.81) | <0.001 |
| Stroke | 1.16 (0.99-1.37) | <0.001 | 1.11 (0.94-1.31) | 0.235 |
| Cancer | 1.95 (1.48-2.55) | <0.001 | 1.75 (1.33-2.30) | <0.001 |
| Peripheral arterial disease | 0.83 (0.66-1.05) | 0.117 | 0.83 (0.66-1.04) | 0.107 |
| Liver cirrhosis | 1.75 (0.93-3.27) | 0.081 | 1.59 (0.84-3.01) | 0.152 |
| **1 year all-cause rehospitalization** | | |  |  |
| Diabetes mellitus | 1.24 (1.13-1.36) | <0.001 | 1.18 (1.07-1.31) | 0.001 |
| CKD | 1.26 (1.15-1.39) | <0.001 | 1.12 (1.01-1.25) | 0.026 |
| COPD | 1.03 (0.91-1.15) | 0.666 | 0.97 (0.86-1.10) | 0.672 |
| Anemia | 1.22 (1.10-1.36) | <0.001 | 1.09 (0.97-1.22) | 0.135 |
| Stroke | 1.12 (1.0-1.25) | 0.046 | 1.04 (0.93-1.17) | 0.483 |
| Cancer | 1.28 (1.04-1.57) | 0.019 | 1.15 (0.93-1.42) | 0.189 |
| Peripheral arterial disease | 1.13 (0.98-1.30) | 0.097 | 1.10 (0.96-1.27) | 0.183 |
| Liver cirrhosis | 1.75 (1.13-2.73) | 0.013 | 1.59 (1.02-2.49) | 0.041 |

*Adjusted for age, sex, educational attainment, marriage, smoking, HR, SBP, LVEF, Hs-cTnT, NT-pro BNP, prior CHD, prior VHD, prior AF

**eTable 2. Sensitivity analysis using IPW to assess the effect of the burden of non-cardiac comorbidities on quality of life**

|  | **KCCQ summary score** | | | |
| --- | --- | --- | --- | --- |
|  | **Baseline** | | **6 month (IPW)** | |
|  | **Unadjusted** | **Adjusted*** | **Unadjusted** | **Adjusted** |
| None | 0 (ref) | 0 (ref) | 0 (ref) | 0 (ref) |
| One vs. None | -3.1 (-4.8,-1.4) | -1.6 (-3.1, 0.0) | -2.4 (-4.1,-0.6) | -0.5 (-2.2,1.2) |
| Two vs. None | -6.1 (-7.9, -4.3) | -3.5 (-5.3, -1.7) | -6.7 (-8.6, -4.7) | -3.2 (-5.2, -1.2) |
| Three plus vs. None | -8.8 (-10.7, -6.9) | -5.4 (-7.4, -3.4) | -11.4 (-13.4, -9.4) | -6.4 (-8.6, -4.2) |

*Adjusted for age, sex, educational attainment, marriage, smoking, NYHA class, HR, SBP, LVEF, Hs-cTnT, NT-pro BNP, BUN, prior CHD, prior VHD, prior AF.

Abbreviations: KCCQ: Kansas City Cardiomyopathy Questionnaire; IPW: Inverse Probably Weighting
